# Temperature and Anæsthesia

**Published:** 1894-03-17

**Authors:** Benjamin Ward Richardson


### TEMPERATURE AND ANÆSTHESIA.

By Sir BENJAMIN WARD RICHARDSON, M.D., F.R.S.

WHILE the active controversy is in progress on the question of the relative effects and value of various anæsthetics, certain general principles influencing the action of anæsthetics are curiously omitted in the controversy. We are told, for instance, that chloroform produces a fatal result during its administration when it affects the respiration. This is the teaching of one school. By another school we are taught that chloroform, when it destroys life, produces its fatal effect through the circulation; and, so far has this kind of antagonism proceeded, that upon it, as if doctrine alone were involved, men pin their faiths, one way or another, without reserve and with entire self-confidence.

Meantime, external influences, which modify so greatly the action of every substance and even of the same substance under varying conditions, are practically ignored. The influences of heat, cold, barometric pressure, condition of the man or animal who is the subject of observation (except so far as actual pathological states are concerned), the solubility of the substance administered, the electrical qualities of the agent being administered, or of the organism receiving it—these, and many other points of greater importance remain forgotten. In a word, the whole question of therapeutical action is narrowed down to one or two particular results, obtained by making experiments, *minus* modifying surroundings. I would like to divert attention for a moment to certain modifying influences, as distinct from naked results springing from experiments in which such influences are set aside.

A simple but instructive illustration comes before me in this way, bearing on the relationships of anæsthesia to temperature. I observed very early in my studies on chloroform, ether, and other vapours, that patients submitted to them exhibited very different symptoms at different seasons of the year, and I observed also that the recorded mortality from chloroform seemed to be marked out by particular periods, so that chloroform death assumed what might almost be called an epidemic type. I named this to Dr. John Snow, who, admitting that there was possibly some importance in the suggestion, because the quantity of vapour of chloroform taken up by the air at different temperatures varied considerably, thought at the same time that any meteorological effect would be wrought through the condition of the person or animal inhaling rather than by any modification of the process of inhalation. If, Snow argued, atmospherical variation caused decisive effects, then every administrator ought to see such effects with so much constancy that there could be no mistake in observation. At the same time, for meeting variations of temperature, he was led to make provision in the inhaler which he used so many times during a long course of years, for an inner bottle, with a surrounding jacket so that he could, if he pleased, by the use of heated water establish a rapidity of vaporisation when the temperature of the air was low. After a while he ceased to utilise this plan on the grounds that it was troublesome, a loss of time, and unnecessary, because he had learned by practice how to regulate the supply of vapour to the patient, under all circumstances, by mere mechanical art and regulation of the mouthpiece valve. He was supported in this view by the fact that although he had had some very narrow escapes, he had never lost a patient from chloroform, after administering it upwards of four thousand times.

Much as I admired my friend, and greatly as I trusted his shrewdness and honesty in observation, I was never convinced that in the matter now under consideration he was strictly correct, and I made a long series of experiments, which placed beyond doubt that he was incorrect, and that even with moderate fluctuations of heat and cold, such fluctuations for example, as occur naturally in our own climate, the character and symptoms of ordinary anæsthesia are distinctly modified. In the way of experiment, I constructed a large glass chamber. At its upper part I stretched a canvas screen over a perforated metal plate for receiving chloroform or other anæsthetic fluid. At its lower part, beneath a perforated stage, I made a regulating surface, through which air, at different temperatures, could be made to pass into the centre or body of the chamber. By this means I could bring the temperature in the centre of the chamber down to 40 deg. Fahr.; or, by changing the lower space so as to make it a warming surface, I could raise the central temperature of the air to 80 deg. Fahr. The apparatus being got into perfect working order, I was able to test the effect of chloroform vapour on various animals, under various conditions of temperature, and also to test the same animal, at different times, at different temperatures, so as to obviate the objection that variety of animal could be a cause of variety of action. Further, by connecting an inhaling tube and mouthpiece with the inside of the body of the chamber I could make the test on the human subject under varying external temperatures. The results of these experiments were so uniform I can have no hesitation in expressing from them that the disturbing influences of heat and cold on the anæsthetic process are marked in a degree which calls for the most serious attention, and which puts the most important reading on some of the experiments which have recently guided, in one or other direction, the opinions of the profession. For example, when a warm-blooded animal was placed in the chamber, with forty grains of chloroform spread on the canvas surface and allowed to diffuse through the air at 40 deg. Fahr., the symptoms of narcotism were invariably prolonged. The first stage was one of considerable excitement; the second stage was attended with shiverings, and in birds—pigeons—with vomiting, partial recovery, relapsing into the second stage, and finally slow passage into the third stage or complete anæsthesia. Seven minutes was often required for production of complete anæsthesia, with fifteen minutes afterwards for recovery in pure air.

When the conditions of the chamber were reversed, when the lower surface was warmed, so that the temperature of the air in the body of the chamber was raised to 80 deg. Fah., and the same quantity of chloroform was diffused through it, the difference in action on the same animals was definite. There was no excitement, no vomiting, little loss of time; in a short interval of two minutes there was gentle sleep, passing at once into deep anæsthesia, the second and troublesome degree of narcotism being passed over altogether. On the human subject the same results were equally well demonstrated.

